# Long-Term Followup with Evaluation of the Surgical and Functional Results of the Ileal Pouch Reservoir in Restorative Proctocolectomy for Ulcerative Colitis

**DOI:** 10.5402/2011/625842

**Published:** 2011-06-16

**Authors:** Ola Røkke, Knut Iversen, Torill Olsen, Sølvi-Mai Ristesund, Geir Egil Eide, Gitta Erika Turowski

**Affiliations:** ^1^Department of Surgery, Akershus University Hospital, N-1478 Lørenskog, Norway; ^2^Faculty of Medicine, University of Oslo, 0316 Oslo, Norway; ^3^Department of Gastrointestinal surgery, Akershus University Hospital, N-1478 Lørenskog, Norway; ^4^Department of Surgery, Diakonissehjemmet Hospital, Haraldsplass, 5021 Bergen, Norway; ^5^Department of Surgery, Haukeland University Hospital, 5021 Bergen, Norway; ^6^Centre for Clinical Research, Haukeland University Hospital, 5021 Bergen, Norway; ^7^Research Group on Lifestyle Epidemiology, Department of Public Health and Primary Health Care, University of Bergen, 5020 Bergen, Norway; ^8^Department of Pathology, Ulleval University Hospital, 0424 Oslo, Norway

## Abstract

*Aims*. Evaluate the early and long term surgical and functional results of the ileal pouch-reservoir (IPAA) in patients with intractable ulcerative colitis. *Material and Methods*. Followup of 134 consecutive patients with W-or J-ileal pouch by diseases-specific and general health (SF-36) questionnaire. In the first 44 patients, early and late followup was performed. *Results*. Followup was performed 7.4 years (0.5–17 years) after construction of W (*n* = 9) and J (*n* = 125) ileal pouch, which had similar results. There were 14.9% early and 43.6% late complications with 12.7% early and 19.5% late reoperations. Protecting loop-ileostomy used in 54 patients (43.9%), did not protect against complications. Thirteen reservoirs (9.8%) were resected (*n* = 8) or deactivated (*n* = 5) due to functional failure. Operation time, postoperative complications and pouchitis were determinators for reservoir failure and reduced quality of life. The functional results at followup of 44 patients at 2.5 years (0.8–6.7 years) and 11.5 years (8.2–19.2 years) were remarkably similar. *Conclusions*. IPAA is a good option for most patients when medication fails. 10% experience failure with inferior quality of life. Protective stoma will not reduce failure rates. After an initial time period, reservoir function will not change over time.

## 1. Introduction

In spite of progress in medical therapy of ulcerative colitis, surgical treatment is still important when medical treatment fail. Ileal pouch-anal anastomosis (IPAA) was introduced in 1978 by Parks and Nicholls [1] and is now the most commonly used surgical technique. The method is safe, with low mortality rates, and most patients avoid permanent ileostomy with good functional results. Some controversies still exist about the type of reservoir, type of anastomosis, the use of protective loop ileostomy, and the risk of malignant development in the residual colon mucosa or reservoir [2]. The morbidity rates are also not without concern. Several series reports on failure rates of 10% and postoperative morbidity rates of 30–50%, with high frequency of reoperations [3–6]. 

The aim of the present study was to describe the surgical and functional results of ileal pouch surgery, to evaluate the effect of temporary diverting ileostomy, to identify possible reasons for reservoir failure, and to perform early and late quality of life measurements in patients with functional and deactivated reservoirs to evaluate time trends in patients treated at one university hospital.

## 2. Materials and Methods

134 consecutive patients with ulcerative colitis treated with restorative proctocolectomy and IPAA at Haukeland University Hospital from 1988 until 2002 were included in the study. The surgical procedures were performed by a limited number of senior surgeons. The quadruple loop W-ileal pouch construction was used in 9 patients (6.7%) during the first years, and the J-ileal pouch construction [7] in 125 (93.3%). The W reservoirs were hand sewn and the J reservoirs were created by suture machines (3 GIA-80) with a length of at least 15 cm (15 to 20 cm). The anastomoses were created by suture instruments with the double stapling technique. The rectal dissection was performed near the rectal wall until 1995. Thereafter, the mesorectum was included in the rectal excision. A temporary diverting ileostomy was used as a routine during the first years, but, from 1993, ileostomy was used only when considered necessary by the operating surgeon. The indications for surgery were benign ulcerative colitis in 129 patients (96.3%), ulcerative colitis with mucosal dysplasia in two patients (1.5%), and ulcerative colitis with adenocarcinoma in three patients (2.2%).

Medical records were reviewed and recorded in a database (SPSS, Illinois, USA). The patients were invited to two followups. The first followup was performed in 1993 of the 48 patients treated until then. This followup was performed by mail. The patients were asked to fill in a specially designed disease-specific questionnaire about their functional status. The questionnaire consisted of 38 specific questions about physical, social, sexual, and food restrictions, occupation, medication, incontinence, and more and 44 patients (92%) responded to this followup. The second followup took place in 2001-2002 at the Department of Surgery by a surgeon and specially trained nurses. All patients were asked to fill in two written questionnaires which they received by mail; one was the same specially designed disease-specific questionnaire used in 1993. Thus it was possible to study potential changes in reservoir function over a nine-year period. The other questionnaire was the Short Form Health Survey questionnaire (SF-36) [8], which records the general quality of life and restrictions in the physical, pain, vitality, social, and mental dimensions. The SF-36 score has been validated in the Norwegian population, and sex- and age-adjusted scores exist for the general population [9]. As many as possible of these questionnaires were completed in collaboration with nurses together with the patient at the followup. Patients who did not attend the followup or submitted incomplete questionnaires received new questionnaires several times until 2006 to make the study as complete as possible. There were, however, some questions in the questionnaire that the patient did not answer. 

The study was approved by the Norwegian Ethical committee.

## 3. Statistics

Gosset's two-sided *t*-test [10] or analyses of variance (ANOVA) was used to test differences between means. Pearson's two-sided chi-square test [11, 12] was used to compare proportions and Kaplan-Meier curves [13], and the log-rank test [14] was used to analyse differences in survival times for functional reservoirs between groups. The prognostic significance of selected factors for reservoir failure was analysed using the Cox proportional hazards regression model [15].

## 4. Results

A total of 134 patients, 77 men (57.5%), and 57 women (42.5%), mean age 42.8 years (17–72 years), were treated with W reservoirs (*n* = 9) or J reservoirs (*n* = 125). The mean age at start of the disease was 27.9 years (3–60 years), and mean age at reservoir construction was 35.6 years (9–69 years). Four patients (3%) had respectively hypertension (*n* = 1), obstructive lung disease (*n* = 2), and primary sclerosing cholangitis (PSC) (*n* = 1); the others were otherwise healthy. Surgery was performed as either 3-stage procedure in 50 patients (37%), 2-stage procedure in 76 patients (57%), or one stage procedure in 8 patients (6%). The mean time from start of the disease until reservoir construction was 7.6 years (0.8–32 years). 

At the first followup in 1993 of the 48 patients operated until then, the mean age was 34.5 years (15–60 years), and mean time from functional reservoir to followup was 2.5 years (0.8–6.7 years). At the second followup in 2002, three of these patients had removed their reservoirs, and 41 of the 45 remaining patients (91%) with functional reservoirs answered the questionnaire. The second followup was attended by 101 patients (75%) and 119 patients (89%) finally responded to the questionnaires. The observation time after reservoir construction for all patients was 7.4 years (0.5–17 years). The observation time with functional reservoir was 6.8 years (1–16 years).

### 4.1. Acute Colectomy

Acute colectomy was performed in 112 patients (84.2%), 64 men (57.1%) and 48 women (42.9%) because of exacerbation of colitis not controlled by medication; 110 (98.2%) patients with ileostomy, two patients (1.8%) with ileorectal anastomosis. 100 (89.3%) of these patients used steroids and 72 patients (64.3%) received antibiotic therapy prior to colectomy. Complications occurred in 13 patients (12.5%) and eight of these (7.2%) needed reoperation (Table 1). There was a significant association between weight loss before acute surgery the and development of complications, especially small bowel obstruction (*P* = .008). Patients with complicated recovery had a longer stay in hospital before operation (25 versus 13 days), but this was not significant (*P* = .071) (Table 1). The patients operated acutely were a little younger (41.6 years (17–69 years)) than the 22 patients (15.8%) with planned operations (48.2 years (30–72 years)) (*P* = .016).

### 4.2. W Versus J Reservoir

The reservoirs were constructed 18 months (3–50 months) after the acute colectomy. The two patients operated with acute colectomy and ileorectal anastomosis were both females, and reservoir constructions were performed 13.5 and 14.5 years after the emergency surgery. The operation time for patients previously operated with colectomy (168.6 minutes (90–378 minutes)) was about the same as in patients not previously operated (152.7 minutes (95–200 minutes)) (*P* = .227), as was the patients' weight and height. The need of blood transfusion was lower (0.2 units SAG (0–7 units)), in patients previously colectomized compared to patients without previously surgery (1.2 units (0–9 units)) (*P* = .003). The time from start of disease until reservoir construction was also shorter for colectomized patients (6.6 years (0.8–24 years)) compared to patients not previously operated (13 years (1.5–32 years)) (*P* = .000).

 The operation time for the hand-sewn W reservoirs (199 minutes (130–230 minutes)) was longer compared to the J reservoirs (167 minutes (90–378 minutes)), but the difference was not statistically significant (*P* = .072). The need of blood transfusions (0 units (0-0 units) versus 0.33 units (0–9 units)) did not differ significantly (*P* = .414).

### 4.3. Early Postoperative Complications

The postoperative complications and reoperations within 30 days after reservoir surgery are shown in Table 2. There were a total of 14.9% early complications with 12.7% reoperations. All complications and reoperations occurred in the group with J-reservoir construction, but the difference was not significant (*P* = .890). In three patients with postoperative bleeding, bleeding vessels were identified and ligated. In one patient, the reservoir was resected due to bleeding and ischemia five days after reservoir construction. Of the eight patients with postoperative small bowel obstruction, one patient was successfully treated conservatively; seven patients needed laparotomy with the division of adherences. One of these, without temporary diverting ileostomy, needed bowel resection with ileostomy. Of the six patients with anastomotic leaks, one was not associated with clinical sepsis and was treated conservatively. Of the other five, four were treated with transanal suture and one with deviating loop ileostomy. In the patient with stomal necrosis, laparotomy showed torquation of the small bowel at the entrance of abdominal wall. Detorquation and stomal revision was performed.

### 4.4. Late Postoperative Complications

There were a total of 43.6% late complications and a 19.5% reoperation rate, with no differences between the groups (Table 3). Of the 35 patients with pouchitis, 31 were treated conservatively. Four developed intractable disease; two were treated with the removal of the reservoir, two with deviating loop-ileostomy. Of the six patients with anastomotic stenosis, five were treated with ambulatory dilatation. One needed hospitalisation and several dilatations in general anaesthesia. Of the five patients with severe diarrhoea and associated faecal incontinence, the reservoir was removed in one patient, and a deviating stoma performed in another. Two patients developed bowel obstruction after W reservoir. Both were treated with the division of adherences; one needed a short bowel resection. Of eight patients with bowel obstruction after J reservoir, all patients were operated with the division of adherences and two needed bowel resections. Six patients had persistent fistula from the anastomoses. All these patients needed defunctioning of the reservoir; four with the removal of the reservoir, and two with deviating loop ileostomy. One patients experienced severe sequela after postoperative leak/pelvic sepsis. The reservoir became stiff with small capacity and was removed. Thus, a total of 13 reservoirs (9.8%) were removed or defunctioned during the observation period due to functional failure (Table 3).

Surgery in three, two, and one stage did not differ with respect to reservoir failure. All eight patients where surgery was performed in one stage still have functional reservoirs. After two and three stage surgery, 68 (89.5%) and 44 (88.0%) of the reservoirs, respectively, were functional at followup (*P* = .588).

**Figure 1:**
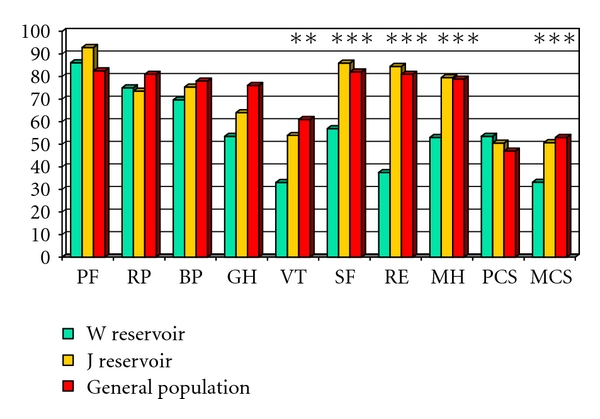
SF-36 scores for patients operated with W reservoir compared to patients with J reservoir; and scores for the normal population. Subscale scores in the Short Form Health Survey questionnaire (SF-36). Higher scores indicate better function. PF equal physical function sum score, RP equal role limitations/physical sum score, BP equal bodily pain sum score, GH equal general health sum score, VT equal vitality sum score, SF equal social function sum score, RE equal role limitation/emotional problems, MH equal mental health sum score, PCS equal physical health summary score, MCS equal mental health summary score. Significances are calculated between J and W reservoirs: **P < .01, ***P < .001.

There were no major functional differences between the W and J reservoirs with regard to frequency of defecation (Table 4). There were, however, some differences in the SF-36 scores (Figure 1). The SF-36 scores of patients with functional J reservoirs were similar to the sex- and age-adjusted values for the general population for all dimensions. In patients with the W reservoir, the vitality sum score, social function sum score, role limitation/emotional problems, mental health sum score, and mental health summary score were significantly lower than the J reservoir. There were no differences in other variables like age at onset of disease, age at reservoir construction, age at followup, gender, subjective reservoir problems, the use of medication, or work-related, sexual, food, physical or social restrictions. The only detectable significant difference among the groups, aside from the SF-36 scores, was the time with functional reservoir for W reservoirs (13.3 years + 1.9 years) and J reservoirs (6.3 years + 3.8 years) (*P* = .014).

### 4.5. Early and Late Followup of Patients with W and J Reservoirs

**Figure 2:**
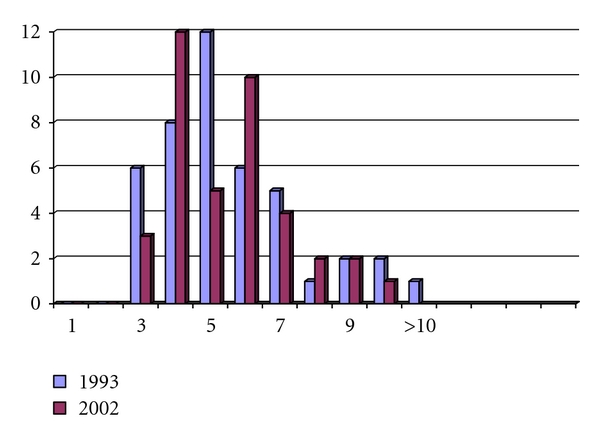
Number of average day-time defecations in 44 patients in 1993 compared to 2002.

**Figure 3:**
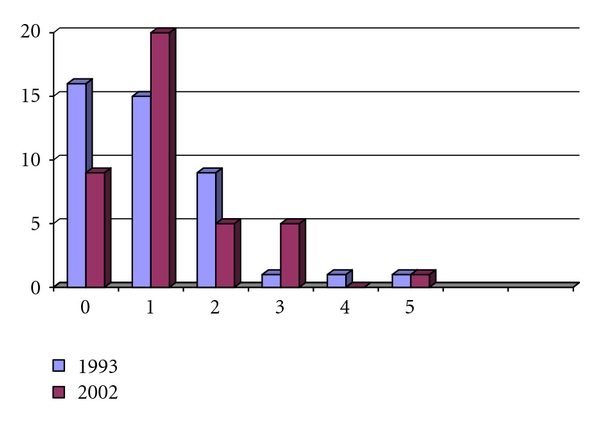
Number of average night-time defecations in 44 patients in 1993 compared to 2002.

At the first followup in 1993 of the 48 patients operated until then, the mean age was 34.5 years (15–60 years). At the second followup in 2002, three of these patients had removed their reservoirs, and 41 of the 45 remaining patients (91.1%) with functional reservoirs answered the questionnaire. The results of the early and late followups are presented in Table 5 and Figures 2 and 3. The observation time for the early followup was 2.5 years (0.8–6.7 years). The observation time for followup of these patients nine years later in 2002 (-2006) was 11.5 years (8.2–19.2 years). The functional results in the followup in 1993 were remarkably similar to the results nine years later, with no significant differences in any of the questions asked! This finding indicates that the results after a certain “adaption period” will remain unchanged.

### 4.6. Protective Loop Ileostomy

**Figure 4:**
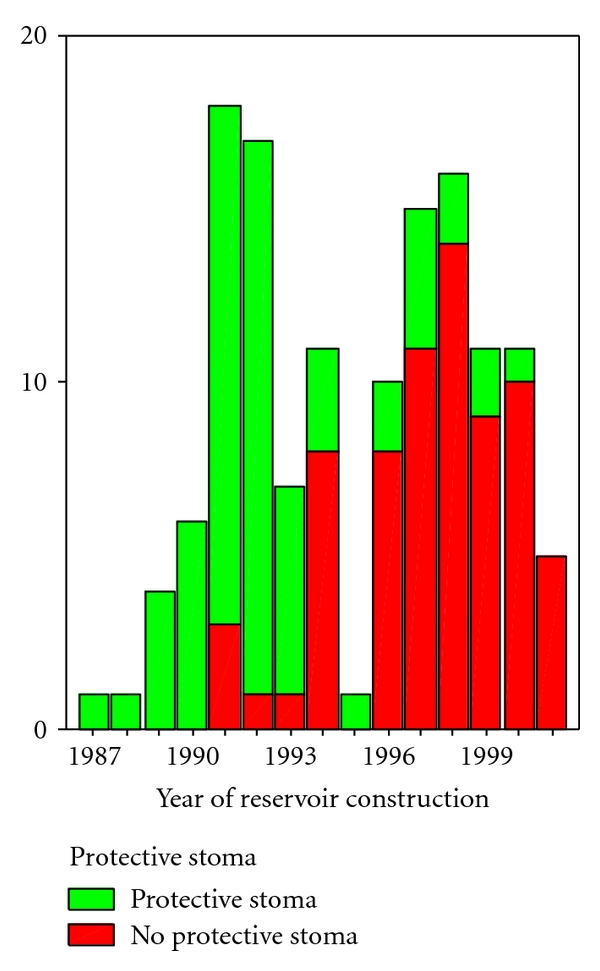
Number of reservoir constructions and the use of diverting stoma during the study period.

Deviating stoma was used in 54 patients (43.9%), but decreasingly during the observation period (Figure 4). There were neither difference in the rates of postoperative complications nor reoperations between patients with or without deviating stoma (*P* = .313), as shown in Table 6. At all, 54 patients (43.5%) with J reservoir experienced late complication during the observation period. 35 patients (26.3%), 18 patients with and 17 patients without deviating stoma, developed pouchitis, six patients (4.9%) (2 versus 4 patients) developed anastomotic stenosis, four patients (3.3%) (2 patients versus 2 patients) developed intractable diarrhoea, eight patients (6.5%) (6 patients versus 2 patients) developed small bowel obstruction, six patients (4.9%) developed fistula from the anastomoses (3 patients versus 3 patients), two patients (1.6%) developed ventral hernia (0 versus 2 patients), and one patient (0.8%) developed necrosis of the reservoir (0 versus 1 patient). One patient (0.8%) without deviating stoma developed urinary retention and needed intermittent catheterisation for two years but has now resumed normal urinary function. One patient (0.8%) without deviating stoma developed intractable diarrhea and fecal incontinence the first two years after operation, but has now regained control of stool with satisfactory consistence. Reoperations in the followup period are shown in Table 7, with no significant differences between the groups (*P* = .306). 

Four of the five patients (80%) reoperated for anastomotic leakage developed reservoir failure at a later stage, the one (100%) without protective stoma and three of the four (75%) with protective stoma.

### 4.7. J-Reservoirs and Failure

**Figure 5:**
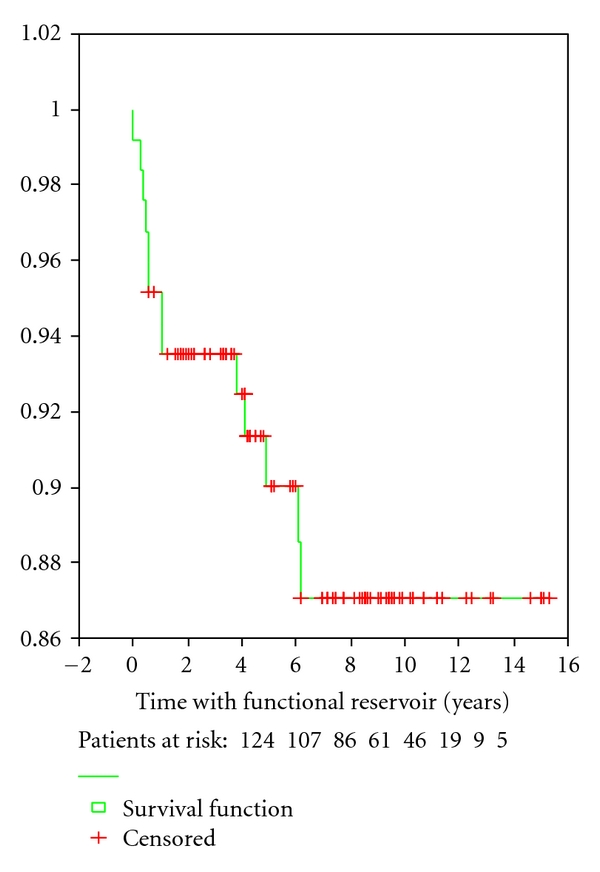
Number of functional J- reservoirs during the observation period.

A total of 125 patients were operated with J reservoir. In one patient, the reservoir was removed postoperatively the 5th day because of bleeding and circulatory disturbances in the reservoir, leaving 124 patients for followup. Failure was determined if the patient had intractable diarrhea, pain, or intractable fistula and would not continue life with the reservoir. Failure occurred in 13 patients (10.4%) after J-reservoir operation (Table 3).  Figure 5 shows a Kaplan-Meier plot of “surviving” reservoirs. In eight patients, the reservoir was removed with permanent ileostomy. In five patients, a loop ileostomy was performed without the removal of the reservoir. Seven of the 13 reservoirs (53.8%) were deactivated within one year.

**Figure 6:**
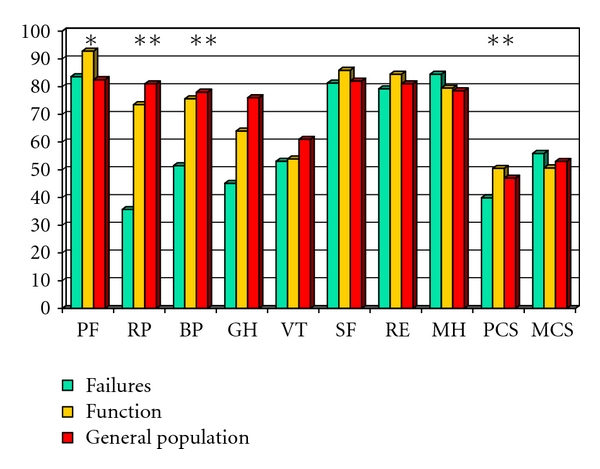
SF-36 scores in patients with functional J reservoirs compared to patients with failed J reservoirs, and scores for the normal population. Subscale scores in the short form health survey questionnaire (SF-36). Higher scores indicate better function. PF equal physical function sum score, RP equal role limitations/physical sum score, BP equal bodily pain sum score, GH equal general health sum score, VT equal vitality sum score, SF equal social function sum score, RE equal role limitation/emotional problems, MH equal mental health sum score, PCS equal physical health summary score, MCS equal mental health summary score. significances are calculated between functional and failed reservoirs: **P < .01, ***P < .001.

Some of the reservoir failures were related to the early postoperative complications. Four of the five patients treated for postoperative leaks developed reservoir failure (80%). None of the patients reoperated for bleeding, small bowel obstruction, and stomal necrosis developed reservoir failure. Nine of the 108 patients (8%) with uneventful recovery developed reservoir failure. We looked into other possible determinants for failure. Operation time for the reservoir procedure (162 minutes in functional reservoir versus 200 minutes in failures), postoperative complications (11.7% in functional reservoir versus 46.2% in failures), postoperative reoperations (10.8% in functional reservoir v. 30.8% in failures), and number of pouchitis (1.63 + 4.64 in functional reservoir versus 8.77 + 18.41) in failures were significant contributors in a univariate analyses. Operation time, postoperative complications (11.7% in functional reservoir versus 46.2% in failures) and number of pouchitis were independent determinants of reservoir failure as shown in Table 8. The effect of reservoir failure on aspects of quality of life is shown in Table 9 and Figure 6. Eight (61.5%) of failures and 103 (93.6%) of the 110 patients with active reservoir answered the questions. The SF-36 scores, especially the physical scores for patients with failures, were significantly lower than scores for patients with functional reservoir. 

## 5. Discussion

IPAA is considered the best surgical option when medicines fail. The quality of life of IPAA patients in long-term followup is similar to that of patients with mild ulcerative colitis or ulcerative colitis in remission [16]. Good quality of life and 95% overall patient satisfaction after IPAA have been reported [17]. In the present study, most patients came to surgery due to acute exacerbation of disease, not controlled by medication. Colectomy was followed by a low complication rate. However, patients with long delay for colectomy and severe weight loss were prone to postoperative complications, especially bowel obstruction, supporting the well-known concept of early surgery in patients in need of colectomy.

The results after W and J reservoirs were quite similar. As only nine patients were operated with W reservoirs, our results must be judged with caution. The quadruple (W) reservoir was originally constructed as an alternative to the J reservoir to achieve larger reservoir volume, and thereby improving the functional results of the J reservoir. Nicholls and Lubowski reported frequency of defecation per 24 hours of 3.3 with night evacuation in 14%, antidiarrhoeal medication in 20% and normal continence in 92% with this reservoir [18]. The relation between pouch size and functional results have been shown in a prospective trial by Nicholls and Pezim [19], comparing three different designs of reservoirs: triple loop, double loop (J reservoir), and quadruple loop (W-reservoir). The J reservoirs were significantly smaller than the other two, and there was an inverse relationship between reservoir volume and defecation frequency. In a prospective randomised trial comparing W and J reservoirs, Selvaggi et al. reported superior functional results of the W reservoir during the “maturation period”, that is, the first year after ileostomy closure, as night-time defecations and the use of antidiarrhoeals were lower after W-reservoirs [20]. However, another prospective controlled trial comparing short (30 cm ileum) or long (40 cm ileum) duplicated (J) versus short (30 cm ileum) or long (40 cm ileum) quadruplicated (W) IPAA showed that the bowel frequency in smaller J reservoirs did not differ significantly from bowel frequency in the bigger W reservoirs. Patients with the large W40, however, had the lowest frequency of the four groups [21]. This is in line with our results, as the bowel frequency after the W reservoir was about the same as after the J reservoir. The reason for lower quality of life of patients with W reservoirs in the vitality, social, emotional, and mental dimensions as measured by the SF-36 score is uncertain. No clinical determinants could be defined. The only difference between the W and J reservoirs was the time with functional reservoir, 13.3 years + 1.9 years and 6.3 years + 3.8 years, respectively. There is a clinical observation that a number of patients operated with reservoirs suffer from chronic fatigue, like in other chronic illnesses, with consequences for quality of life. It is possible that this is accentuated over time with reservoirs and leads to lower quality of life. 

In the present study, the quality of life of patients with IPAA was remarkably stable. The proportion of patients with work, social, sexual, food, and other restrictions or improvements was the same at the early followup two years (1 year–6 years) after operation and at the later followup ten years (8 years–19 years) after the operation. Three patients had their reservoir deactivated during the years between the first and second followup. They did not fill in the questionnaire, and they are not counted. These patients would be expected to contribute to a lower quality of life at the late followup, as is shown for other patients with reservoir failures in the present study. The functional stability over years may be explained by a study of Harms et al. [22]. They reported on 109 W reservoirs and measured static compliance 2 and 12 months after ileostomy takedown and after 3 years in 25 patients. They demonstrated a decrease in 24-hour stool frequency from 2 months to 1 year, and a simultaneous increase in reservoir compliance. Thereafter, no significant change occurred. 

The question of protective stoma is important. Several surgeons prefer a protective ileostomy during the time period for anastomotic healing to reduce the effects of suture line defects and prevent pelvic sepsis, as anastomotic failure after restorative proctocolectomy is associated with a high rate of pouch failure [23]. They argue that leaks will be not so disastrous consequences, and that the reservoir will not be so damaged under the protection of a stoma. However, a protective stoma also has complications, and a rate of 8.5% of serious complications of ileostomy in need of laparotomy has been reported [24]. In Norway, like in most countries in the western world, the body mass index (BMI) is increasing in our population. In patients with high BMI, it may sometimes be difficult to construct a safe protecting loop ileostomy without extra traction on the reservoir. In some of our patients, the construction of a nice protective stoma was impossible due to the combination of short mesentery and high BMI. In a study of Gorfine et al. on J reservoirs, the results of IPAA in 69 patients with protective stoma were compared to 74 patients without deviating stoma. There were 6% suture line defects in both groups. Patients without ileostomy had fewer postoperative complications, fewer episodes of bowel obstruction, fewer instances of reexploration and totally fewer days in hospital [25]. In a study of one-stage (*n* = 57) and two-stage (*n* = 114) IPAA by Heuschen et al., the proportion of patients without complications was higher and the frequency of late complications were lower after one-stage procedure. Early complications, pouch-related septic complications, duration of surgery, blood loss, need of transfusions and hospital stay were the same [26]. We performed eight one-stage proctocolectomy with reservoirs without failures. Thus, this may be an option in selected patients. 

Septic complications related to the IPAA have been reported in up to 16% of patients. In a study of 51 patients with IPAA-related sepsis, sinuses or fistula from the anastomoses, Gorfine et al. could find no difference between success rates of reparative procedures in diverted and nondiverted patients (29.7% versus 20.8%) [23] and reported that pouch function could be retained in 56.9% of their patients. During the second half of our study period, we decided not to use protective ileostomy as a routine, mainly due to the morbidity of ileostomy and the need for a second hospitalisation and operation. This change did not increase the total complication rates. There were one anastomotic leak in the group without protective stoma, and this reservoir could not be saved. With protective stoma, four leaks occurred, and one of these reservoirs could be saved. The numbers are, however, too small for conclusions. 

The results of IPAA in our series are similar to others, with 30–50% complications and 10% failures. The main late problems were small bowel obstruction and reservoir dysfunction due to sequelae of anastomotic leaks, pouchitis or diarrhoea. These are also complications encountered by others [27]. These results are not without concern. To estimate the functional results, standardized quality of life measurements are important. Many of our patients had excellent results; some has less satisfactory results, especially patients with reservoir failures. Some of these failures may be explained by postoperative complications, leaks, and postoperative pouchitis, but most were unpredictable. Coffey et al. assessed quality of life after IPAA, and revealed that 95.3% complained of some form of dietary restriction, that pouchitis gave poorer quality of life, and that parous women had the lowest quality of life [28]. In the present study, we could also demonstrate a much higher incidence of various restrictions after reservoir failures. The SF-36 scores also revealed that failures specifically reduced the physical dimension and pain of quality of life but did not influence the mental or emotional dimensions. 

A recent meta-analysis of 9317 patients showed a pouch failure rate of 6.8% rising to 8.5% in cases with followup of more than 60 months. Severe, mild and urge faecal incontinence occurred in 3.7%, 17%, and 7.3%. The authors state that current techniques for restorative surgery after proctocolectomy are associated with nonnegligible complication rates and leave room for improvement and continuation of development of alternative procedures [6]. 

Some large series report better results. The Cleveland Clinic reported a pouch failure rate of 3.4%, which is a low rate compared to other large series with failure rates of 8%–11% [4, 5]. They suggest that careful selection of patients and attention to surgical details and postoperative followup, as well as the frequent use of double-stapled IPAA may cause their good results [29]. They also report on good quality of life. Others also report on low pouch failure rates and high (90%–95%) long-term patient satisfaction [3, 17, 30].

## 6. Conclusion

IPAA is a good option for many patients with severe ulcerative colitis when medication fails. Surgical pouch-construction is associated with 40% morbidity and 10% failure rates. J reservoirs and W reservoirs have similar results. Protective stoma will not reduce failure rates. After an initial period of time, there is little change in reservoir function. Failures are associated with a decreased quality of life.

## Figures and Tables

**Table 1:** Complications and reoperations after acute surgery and association between factors and complications after acute colectomy in 112 patients with colectomy due to acute exacerbation of ulcerative colitis.

	Complications *n* (%)	Reoperations *n* (%)	Time from start of disease to colectomy (months) mean (SD)	Time in hospital before colectomy (days) mean (SD)	Weight loss before operation (kg) mean (SD)
None	98 (87.5)	103 (92.0)	62 (66)	13 (10)	3.6 (4.8)
Small bowel obstruction	6 (5.4)	6 (5.4)	45 (76)	25 (18)	10.5 (11)
Wound infection	5 (4.5)	0 (0.0)	62 (78)	7 (4)	4.3 (2.1)
Pelvic abscess	1 (0.9)	1 (0.9)	42	21	17.0
Stoma problems	1 (0.9)	1 (0.9)	84	9	—

*P*-value*	—	—	0.971	0.071	0.008

*ANOVA

**Table 2:** Early complications within 30 days and reoperations after reservoir surgery in 134 patients with W or J reservoirs.

	W-reservoir *N* = 9		J-reservoir *N* = 125	
	Complications	Reoperations	Complications	Reoperations
	*n* (%)	*n* (%)	*n* (%)	*n* (%)
None	9 (100)	9 (100)	105 (84.0)	108 (85.6)
Bleeding			4 (3.2)	4 (3.2)
Anastomotic leaks			6 (4.8)	5 (4.0)
Small bowel obstruction			8 (6.4)	7 (5.6)
Stomal necrosis			1 (0.8)	1 (0.8)
Diarrhoea			1 (0.8)	0 (0.0)

**Table 3:** Late complications and reoperations after reservoir surgery in133 patients with W or J reservoirs (one J reservoir removed postoperatively).

	W-reservoir *N* = 9		J-reservoir *N* = 124	
	Complications	Reoperations	Complications	Reoperations
	*n* (%)	*n* (%)	*n* (%)	*n* (%)
None	5 (55.6)	7 (77.8)	70 (56.5)	100 (80.8)
Pouchitt	1 (11.1)	0 (0.0)	34 (27.4)	4 (3.2)
Anastomotic stenosis	0 (0.0)	0 (0.0)	6 (4.8)	1 (0.8)
Diarrhoea	0 (0.0)	0 (0.0)	5 (4.0)	2 (1.6)
Small bowel obstruction	3 (33.3)	2 (22.2)	8 (6.5)	8 (6.4)
Fistula from the anastomosis	0 (0.0)	0 (0.0)	6 (4.8)	6 (4.8)
Ventral hernia	0 (0.0)	0 (0.0)	2 (1.6)	2 (1.6)
Failures				
(i) Postoperative removal	0 (0.0)	0 (0.0)	1 (0.8)	(0.8)
(ii) Late removal	0 (0.0)	0 (0.0)	0 (0.0)	(6.4)
(iii) Late deviating stoma	0 (0.0)	0 (0.0)	0 (0.0)	5 (4.0)

**Table 4:** Faecal frequency with functional W and J reservoirs.

	W-reservoir	J-reservoir	*P*-value*
	*N* = 9	*N* = 111	
	mean (min-max)	mean (min-max)	
Faecal frequency best days	3.5 (3–7)	4.8 (1–15)	.106
Faecal frequency average days	5.1 (4–7)	6.0 (2–13)	.227
Faecal frequency worst days	8.8 (4–10)	10.4 (4–30)	.226
Faecal frequency best nights	0.3 (0–3)	0.5 (0–3)	.044
Faecal frequency average nights	0.8 (0–3)	1.4 (0–6)	.087
Faecal frequency worst nights	2.1 (1–3)	3.6 (0–12)	.444

* *t*-test

**Table 5:** Functional results at early and late followup of the first 48 patients with IPAA.

	Early followup 1993	Late followup 2001–2006	*P*-value*
	*n/N* (%)	*n/N* (%)	
Married	31/44 (70.5)	32/42 (76.2)	.548
Work restrictions	15/44 (34.1)	10/40 (25.0)	.363
Social restrictions	5/44 (11.4)	8/39 (20.5)	.252
Social improvement	9/44 (20.5)	8/41 (20.5)	.914
Sexual restrictions	6/42 (14.2)	4/41 (9.8)	.579
Sexual improvement	11/42 (25.0)	6/41(14.6)	.192
Food restrictions			
(i) Eat food to make stool thicker	6 /44 (13.6)	8/41 (19.5)	.466
(ii) Avoid food that make stool thinner	18/44 (40.9)	23/40 (57.5)	.129
(iii) Avoid food that creates other problems	18/44 (40.9)	16/40 (40.0)	.932
Fecal incontinence often/sometimes	7/44 (15.9)	4/41 (8.8)	.398
Uses diaper often/sometimes	10/44 (22.8)	10/41 (24.4)	.857
Regretted reservoir often/sometimes	8/44 (18.2)	5/41 (12.2) ^#^	.875
Regretted reservoir often/sometimes	—	8/41 (19.5) ^#^	—
Reservoir failures	0/44 (0.0)	3/44 (6.8)	—
Observation time (years)	2.4 (1–6.6)	10.8 (8.3–19.2)	—

*Chi square test.

^#^ *n* = 5: not including patients with failures; *n* = 8: including patients with failures.

**Table 6:** Early postoperative complications (within 30 days) and reoperations after reservoir surgery in 125 patients with J reservoir with or without protective loop ileostoma.

	J reservoir with protective ileostoma		J reservoir without protective ileostoma	
	*N* = 55		*N* = 70	
	Complications	Reoperations	Complications	Reoperations
	*n* (%)	*n* (%)	*n* (%)	*n* (%)
None	45 (81.8)	47 (85.5)	60 (85.7)	61 (87.1)
Bleeding	2 (3.6)	2 (3.6)	2 (2.9)	2 (2.9)
Anastomotic leaks	5 (9.1)	4 (7.2)	1 (1.4)	1 (1.4)
Small bowel obstruction	2 (3.6)	1 (1.8)	6 (8.6)	6 (7.1)
Stomal necrosis	1 (1.8)	1 (1.8)	0 (0.0)	0 (0.0)
Diarrhoea	0 (0.0)	0 (0.0)	1 (1.4)	0 (0.0)

**Table 7:** Late reoperations in 124 patients with and without protective loop ileostoma after J-reservoir reconstruction.

	J reservoir with protective ileostoma	J reservoir without protective ileostoma	All
	*N* = 54	*N* = 70	*N* = 124
	*n* (%)	*n* (%)	*n* (%)
None	41 (75.9)	59 (84.3)	100 (80.6)
Small bowel obstruction	6 (11.2)	2 (2.9)	(6.6)
(i) Bowel resection	1 (1.9)	1 (1.4)	(1.6)
(ii) Division of adherences	5 (9.3)	1 (1.4)	6 (4.8)
Reservoir failure	6 (10.9)	7 (10.0)	10.5)
(i) Removal of reservoir + ileostoma	4 (7.4)	4 (5.7)	8 (6.6)
(ii) Diverting stoma	2 (3.7)	3 (4.3)	5 (4.0)
Ventral hernia: hernioplasty	0 (0.0)	2 (2.9)	2 (1.6)
Anastomotic stricture: dilatation	1 (1.9)	0 (0.0)	1 (0.8)

**Table 8:** Factors of importance for J reservoir failure.

	Univariate analyses	Multivariate analyses ^C^	
	*P*	Hazard ratio (95% CI)	*P*
Age at followup (years)	.757 ^t^	1.00 (0.93, 1.08)	.958
Time from start of UC to reservoir (days)	.342 ^t^	1.00 (0.99, 1.00)	.194
Operation time (minutes)	.020 ^t^	1.01 (1.00, 1.02)	.01
Protective stoma (no = 1, yes = 2)	.793 ^lr^	0.91 (0.20, 4.06)	.898
Postoperative complications (no = 1, yes = 2)	.0004 ^lr^	29.86 (4.00, 223.25)	.001
Postoperative reoperations (no = 1, yes = 2)	.0287 ^lr^	0.21 (0.28, 1.50)	.118
Number of pouchitis (0–50)	.000 ^t^	1.08 (1.04, 1.13)	.000

lr: log rank, t: *t*-test, C: Cox-regression.

**Table 9:** Functional results in patients with active and removed or deactivated J reservoirs.

	Functional reservoir	Removed or deactivated reservoir with stoma	*P* value*
	*n/N* (%)	*n/N* (%)	
Married	80/102 (78.4)	6/8 (75.0)	.976
Work restrictions	34/102 (33.3)	3/7 (42.9)	.607
Food restrictions	75/100 (75.0)	6/7 (85.7)	.523
Physical restrictions	19/69 (27.5)	2/4 (50.0)	.335
Social restrictions	28/97 (28.9)	4/6 (66.7)	.052
Sexual restrictions	17/102 (16.7)	3/6 (50.0)	.041
Faecal incontinence often/sometimes	19/102 (18.6)	—	—
Uses diaper often/sometimes	19/102 (18.6)	—	—
Regretted reservoir often/sometimes	13/101 (12.9)	4/7 (57.2)	.000

*Chi square test.
